# Efficient genome editing in wild strains of mice using the *i*-GONAD method

**DOI:** 10.1038/s41598-022-17776-x

**Published:** 2022-08-15

**Authors:** Yuji Imai, Akira Tanave, Makoto Matsuyama, Tsuyoshi Koide

**Affiliations:** 1 Mouse Genomics Resource Laboratory, National Institute of Genetics, Mishima, 411-8540 Japan; 2 Laboratory for Mouse Genetic Engineering, RIKEN Center for Biosystems Dynamics Research, Osaka, 565-0871 Japan; 3 Division of Molecular Genetics, Shigei Medical Research Institute, Okayama, 701-0202 Japan; 4 Department of Genetics, SOKENDAI (The Graduate University for Advanced Studies), Mishima, 411-8540 Japan

**Keywords:** Genetics, Biological techniques, Genetic engineering

## Abstract

Wild mouse strains have been used for many research studies, because of the high level of inter-strain genetic and phenotypic variations in them, in addition to the characteristic phenotype maintained from wild mice. However, since application of the current genetic engineering method on wild strains is not easy, there are limited studies that have attempted to apply gene modification techniques in wild strains. Recently, *i*-GONAD, a new method for genome editing that does not involve any ex vivo manipulation of unfertilized or fertilized eggs has been reported. We applied *i*-GONAD method for genome editing on a series of wild strains and showed that genome editing is efficiently possible using this method. We successfully made genetically engineered mice in seven out of the nine wild strains. Moreover, we believe that it is still possible to apply milder conditions and improve the efficiencies for the remaining two strains. These results will open avenues for studying the genetic basis of various phenotypes that are characteristic to wild strains. Furthermore, applying *i*-GONAD will be also useful for other mouse resources in which genetic manipulation is difficult using the method of microinjection into fertilized eggs.

## Introduction

Wild mice exhibit differences of phenotypes as compared to laboratory mice, including in terms of behavior, immunological traits, infectious disease, cancer, microbiota, etc*.*^1–5^. A series of wild strains established from wild-captured mice serves as a valuable resource for many studies. Due to the high levels of genetic differences among wild strains, it is expected that there will be a large level of phenotypic differences in them as well. In addition, wild strains are not exposed to deliberate attempts of domestication and maintained in low number of generations of breeding under artificial conditions, before and during establishing strains^6–8^. Therefore, mice of these strains still exhibit behavior that is at least partially characteristic of wild mice, such as high level of responsiveness to handling, higher anxiety levels, stress sensitivity, and intermale aggression^7,9^.

Previous studies have reported that the species, *Mus musculus*, is composed of at least three major subspecies groups, Musculus, Domesticus, and Castaneus, based on their genetic profiles, such as polymorphisms in biochemical markers, mitochondrial DNA, and nuclear genes^8,10–12^. Although, further taxonomic subdivisions into many different subspecies have been applied based on their geographical distribution and morphological characteristics, these local subspecies have been categorized as deviants from the major subspecies groups, because of the distinct genetic differences in them that have not been identified among these subdivided taxonomic subspecies.

Several researchers have carried out devoted attempts and successfully established inbred strains, after at least 20 generations of sister-brother mating among wild mice captured at different places of the world^10–13^. At the National Institute of Genetics (NIG), Japan, Moriwaki and his colleagues established a series of strains and nine wild strains, PGN2/Ms (PGN2), BFM/2Ms (BFM/2), NJL/Ms (NJL), BLG2/Ms (BLG2), CHD/Ms (CHD), SWN/Ms (SWN), KJR/Ms (KJR), MSM/Ms (MSM), and HMI/Ms (HMI), which are now available as genetic resources for biomedical and genetic research, including studies in immunology, cancer, developmental biology, and behavior^6,14,15^. A series of these strains is called the Mishima battery^16^.

Behavioral studies using wild strains in the Mishima battery showed high level of behavioral diversity among strains^6,16–20^. Further genetic analysis identified genetic loci associated with home-cage activity^21,22^, sensitivity to capsaicin^23^, bitter taste sensitivity^24^, social behavior^25^, aggressive behavior^26^, and tameness^27^. In order to show association of candidate genes and these behaviors, it is useful to establish genetically engineered mice based on these wild strains. However, there are a limited studies that have carried out genetic engineering on the wild strains, due to a lack of information about the appropriate methods for gene-targeting or in vitro manipulation of eggs and fertilized eggs, compared to those used in the laboratory strains^28–31^. Previously, Clustered Regularly Interspaced Short Palindromic Repeats/CRISPR associated protein 9 (CRISPR/Cas9) genome editing method using microinjection under in vitro conditions was applied to one of the wild strains, MSM, which has originated from Japanese wild mice, to successfully generate a tyrosinase knock-out mice^31^. Although genome editing was successful in some studies, it requires modification in the method used for preparation and manipulations of eggs, as compared to the commonly used methods^29,30^, and faces the bottleneck of high technical difficulty. These difficulties in terms of in vitro manipulation of eggs and fertilized eggs in wild strains serve as a limitation in the use of wild strains for a variety of genetic studies in many research fields.

Recently, a new method, Genome editing via Oviductal Nucleic Acids Delivery (GONAD), which does not involve any ex vivo manipulation of unfertilized or fertilized eggs has been reported for application in genome editing^32^. In this method, CRISPR guide RNA (gRNA) and Cas9 RNA, which are injected into the oviduct right before, are introduced into fertilized eggs by means of electroporation, through the whole oviduct. Further study showed that using CAS9 protein in place of the RNA improved the efficiency of genome editing from approximately 25%^32^ to 50–100% in knock-out and 15–40% in knock-in; this was reported as a modified method known as *improved*-GONAD (*i*-GONAD)^33^. Since ex vivo handling of unfertilized or fertilized eggs is not required in GONAD and *i*-GONAD, this method serves to be highly efficient not only for laboratory mice, but also for rats^34^, hamsters^35^, and other mammal species in which reproductive technology has not been developed well.

These results implied the possibility of applying genome editing for a variety of wild mouse strains by using the *i*-GONAD method. Genome editing in wild strains will open a new approach, in which gene function in wild strains are examined directly by means gene knock-out and knock-in using wild strains that exhibit a distinguishable phenotype. In this report, we first analyzed efficiencies of in vitro fertilization (IVF) and embryo transplantation into pseudopregnant females, to examine how the efficiencies of manipulations are different from those in the standard laboratory strain. Next, in order to investigate the usefulness of the *i*-GONAD method for wild strains, we conducted genome editing using this method. Finally, because some strains still showed low levels of birth rate, we modified the condition of the electroporation, to improve the efficiencies for obtaining genetically edited mice.

## Results

### Analysis of IVF efficiencies in the wild strains

**Figure 1:**
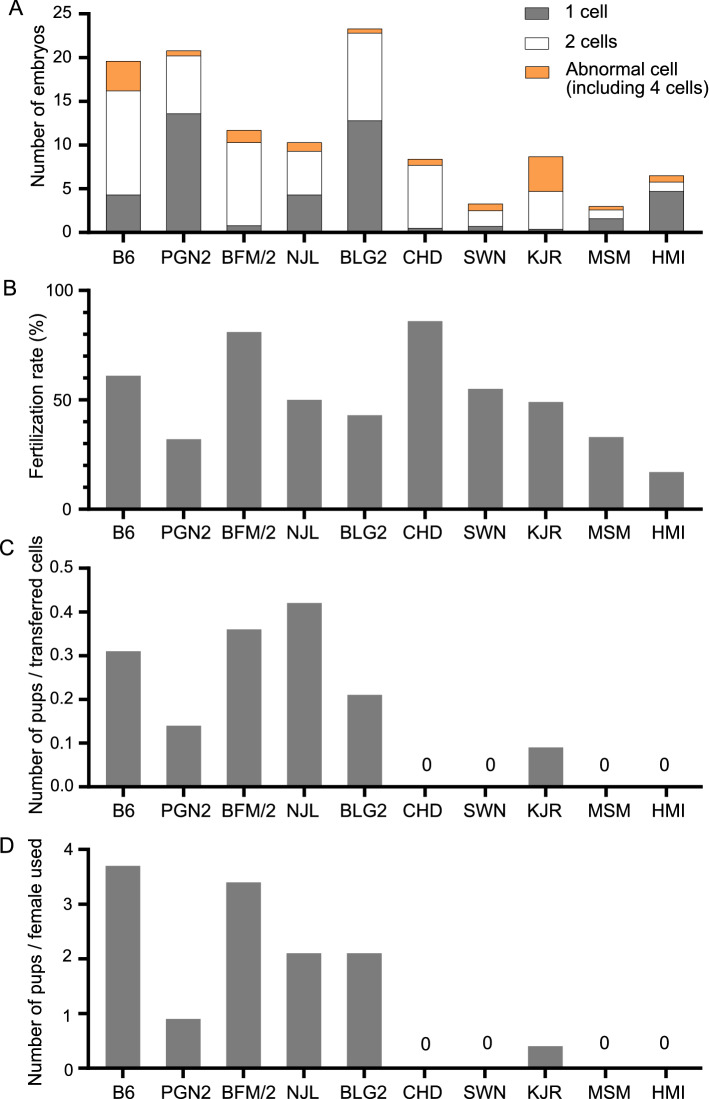
Comparison of the results of the IVF experiments among the B6 strain and nine wild strains. (**A**) Average number of embryos per female, as well as composition of normal embryos (2-cell), non-developing 1-cell eggs, and abnormal embryos, after overnight incubation following IVF. (**B**) Fertilization rates after IVF were calculated by dividing the number of 2-cells by total number of eggs. (**C**) The mean number of newborn pups per transferred cell. (**D**) The mean number of pups per female that was used to collect unfertilized eggs for IVF in each strain.

In order to examine efficiencies of manipulation of eggs and fertilized eggs in the cultured condition, we conducted IVF experiments followed by embryo transfer into pseudopregnant females, using nine wild strains and one laboratory strain C57BL/6JJcl (B6) (Table 1). After IVF and overnight incubation, we sorted the eggs/embryos and counted the numbers of 1-cell eggs (unfertilized, arrested, or delayed development), 2-cell embryos (normal development), 4-cell embryos, and abnormal eggs with morphological abnormalities (Fig. 1A and Table 2). In the BLG2 and PGN2 strains, larger number of eggs and eggs per female were obtained from the female mice than those in the B6 strain, while the other wild strains displayed lesser of these values than the B6 strain. Two strains, SWN and MSM, showed extremely low number of eggs per female, 3.3 and 3.0, respectively. We calculated the fertilization rates by dividing the number of 2-cells by the number of total eggs (Fig. 1B and Table 2). CHD and BFM/2 strains exhibited higher fertilization rates than the B6 strain. On the other hand, the HMI strain displayed an extremely low fertilization rate, of 0.15.

**Table 1:** List of strains used in the study.

Origin	Strain name	Subspecies group	Taxonomic subspecies	Place of collection	Introduction year
Laboratory strain	C57BL/6JJcl	Domesticus			
Wild strain	PGN2/Ms	Domesticus	*M.m.domesticus*	Pigeon, Ontario, Canada	1979
	BFM/2Ms	Domesticus	*M.m.brevirostris*	Montpellier, France	1980
	NJL/Ms	Musculus	*M.m.musculus*	Northern Jutland, Denmark	1980
	BLG2/Ms	Musculus	*M.m.musculus*	General Toshevo, Bulgaria	1981
	CHD/Ms	Musculus	*M.m.gansuensis*	Chendu, China	1981
	SWN/Ms	Musculus	*M.m.musculus*	Suwon, Korea	1984
	KJR/Ms	Musculus	*M.m.musculus*	Kojuri, Korea	1984
	MSM/Ms	Musculus	*M.m.molossinus*	Mishima, Japan	1978
	HMI/Ms	Castaneus	*M.m.castaneus*	Heimei, Taiwan	1986

**Table 2:** Comparison of efficiencies of IVF experiments between wild and laboratory strains.

Strain	Number of donors	Total eggs	Eggs per female	2cell	1cell	4cell	Abnormal cells	Fertilization rate	Transferred 2 cell embryos	Born	Number of recipient females	New born rate
B6	10	186	18.6	119	43	0	34	0.64	119	37	4	0.31
PGN2	10	208	20.8	66	136	0	6	0.32	66	9	2	0.14
BFM/2	10	117	11.7	95	8	0	14	0.81	95	34	4	0.36
NJL	10	101	10.1	50	43	0	10	0.50	50	21	3	0.42
BLG2	10	233	23.3	100	128	0	5	0.43	100	21	4	0.21
CHD	10	84	8.4	72	5	0	7	0.86	72	0	3	0.00
SWN	10	33	3.3	18	7	1	7	0.55	18	0	1	0.00
KJR	10	107	10.7	43	4	0	40	0.40	43	4	2	0.09
MSM	10	30	3.0	10	16	0	4	0.33	10	0	2	0.00
HMI	10	65	6.5	10	47	5	2	0.15	14	0	2	0.00

To examine the efficiencies of birth after embryo transfer, we analyzed the number of newborn pups in each strain at the day of birth. No pups were obtained in the CHD, SWN, MSM, and HMI strains. Newborn ratios were calculated by dividing the number of newborn pups by the number of transferred 2-cell embryos (Fig. 1C and Table 2). BFM/2 and NJL strains exhibited newborn rates of 0.36 and 0.42, respectively, which were higher than that of the B6 strain, which was 0.31. In five strains out of the nine wild strains, that is CHD, SWN, KJR, MSM, and HMI, the newborn rates were less than 0.10, thus indicating that obtaining progeny after in vitro manipulation of eggs or embryos derived from these strains is extremely difficult using the current methods. Number of pups born per female used for collecting eggs was less than 1 in 6 strains, that is PGN2, CHD, KJR, MSM, SWN, and HMI (Fig. 1D and Table 2), again indicating that in vitro manipulation of eggs or embryos is highly difficult in these strains. These results indicated that genome editing using microinjection following in vitro manipulation of fertilized embryos faces major technical problem due to the low efficiency of birth rate after embryo transplantation in most strains. Accordingly, we decided to apply a different method for genome editing instead of the IVF method.

### Application of i-GONAD on wild strains

**Figure 2:**
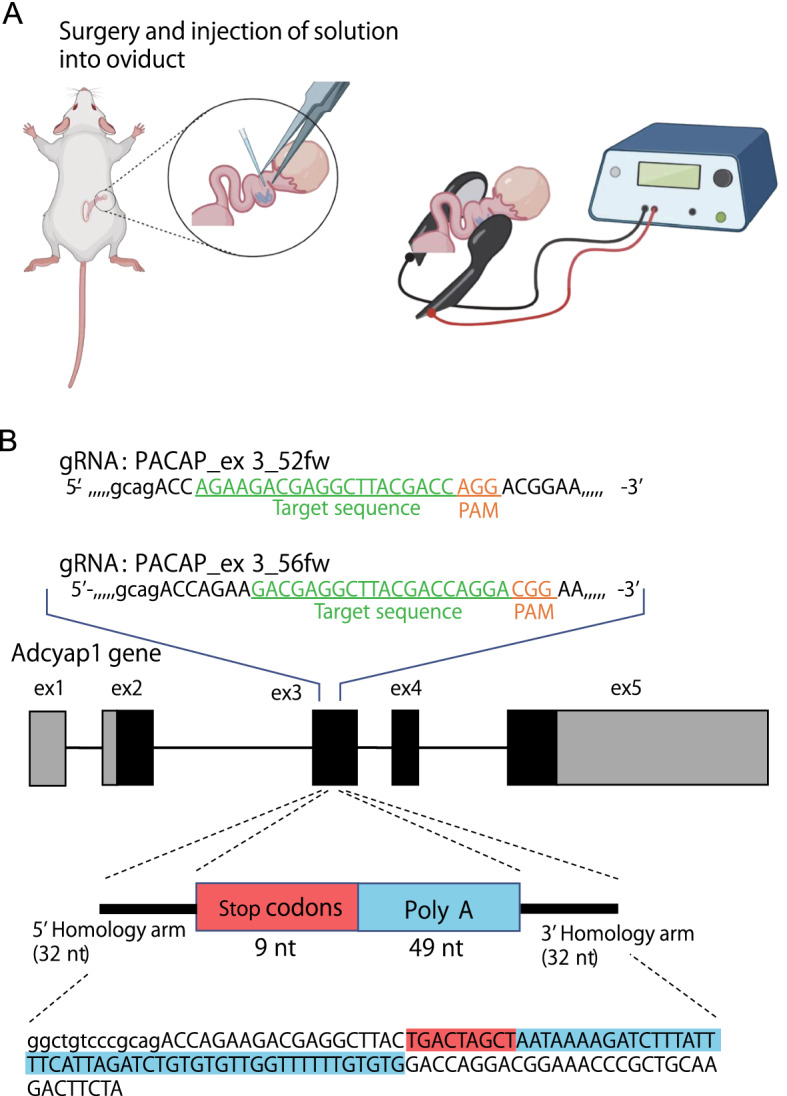
Overview of genome editing with the *i*-GONAD method. (**A**) An illustration of the procedure for the *i*-GONAD method. (**B**) Structure of the *Adcyap1* gene and a design of the ssODN for knock-in of a null mutation by means of genome editing. In the *Adcyap1* gene, filled boxes indicate exons, while black boxes indicate coding regions. The red box and sequence highlighted in red indicate triple stop codons in three coding frames. The last stop codon is overlapped with the following polyadenylation (poly-A) signal. The blue box and sequence highlighted in blue indicate the poly-A signal. Lowercase letters indicate the intron sequence, while uppercase letters indicate the exon sequence.

**Figure 3:**
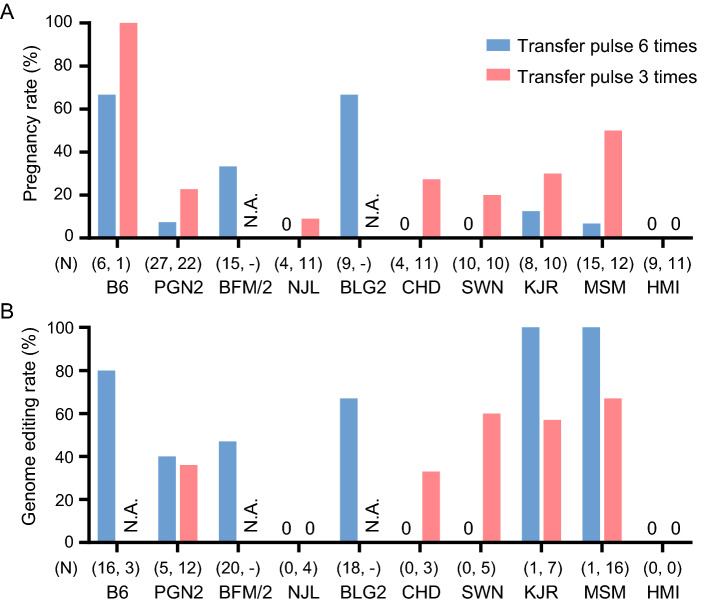
Comparison of efficiencies between the methodologies of 6 times transfer pulse (TP:6) and 3 times transfer pulse (TP:3) in the *i*-GONAD experiments. N; number of pups obtained in the *i*-GONAD experiments in each strain. (**A**) Overview of the pregnancy rates between TP:6 and TP:3. N.A., not analyzed. (**B**) Efficiencies of genome editing upon application of TP:6 and TP:3. The efficiencies were calculated by dividing the number of genome-edited pups by the number of all the sampled pups in each strain. The B6 strain and seven wild strains that displayed low pregnancy rates (< 20%) were further examined with the TP:3 condition.

In order to see how genome editing using *i*-GONAD method helps produce genetically engineered mice in wild strains, we conducted *i*-GONAD with 6 times transfer pulse (TP:6) in all the strains (Fig. 2A). Genome editing was designed to introduce triple stop codons in three coding frames in the exon 3 of *Adcyap1*, a gene that encodes the neuropeptide, Pituitary Adenylate Cyclase-Activating Polypeptide (PACAP) (Fig. 2B). Because PACAP is associated with stress response, introducing a mutation in this gene in wild strains may be useful for future work. As shown in Table 3, the BLG2 strain showed relatively high pregnancy rate (67%), at a similar level as that seen in the B6 strain (67%) (Fig. 3A). Four strains, NJL, CHD, SWN, and HMI, did not give birth at all, while three strains, PGN2, KJR, and MSM, showed extremely low rates (< 15%). BFM/2 strain showed relatively moderate level of pregnancy rates (33%). Number of pups per female was reasonably good in BFM/2 (1.33) and BLG2 (2.00), as compared to that in B6 (2.66). However, the other strains, PGN2, NJL, CHD, SWN, KJR, MSM, and HMI, showed extremely low numbers of pups per female, of less than 0.20.

**Table 3:** Efficiencies of genome editing upon application of *i*-GONAD with 6 transfer pulses (TP:6).

Origin	Strain	Number of females	Pregnancy	Number of pups	Pups/female	MU	KI	Genome-editing efficiency
Laboratory	B6	6	4 (66.7%)	16	2.66	8	4	12/15 (80.0%)
Wild	PGN2	27	2 (7.4%)	5	0.19	2	0	2/5 (40.0%)
	BFM/2	15	5 (33.0%)	20	1.33	6	2	8/17 (47.1%)
	NJL	4	0	0	0.00			
	BLG2	9	6 (66.7%)	18	2.00	6	6	12/18 (66.7%)
	CHD	4	0	0	0.00			
	SWN	10	0	0	0.00			
	KJR	8	1 (12.5%)	1	0.13	1		1/1 (100%)
	MSM	15	1 (6.7%)	1	0.07		1	1/1 (100%)
	HMI	9	0	0	0.00

To assess the efficiency of genome editing, we genotyped all the samples obtained from the pups and analyzed the genome editing rates by dividing the number of genome-edited pups by the number of all the sampled pups in each strain (Fig. 3B and Table 3). The *i*-GONAD method with TP:6 was able to generate multiple genome-edited mice in the PGN2, BFM/2, and BLG2 strains, as well as the laboratory B6 strain. Genome sequences of the editing sites in each pup were analyzed to clarify whether the genome editing sites were knocked in precisely or were mutated with unexpected sequence changes (Supplementary materials S1). The genome-edited mice in each of the B6, BFM/2, and BLG2 strains included both mutation (MU) at the target site, such as insertions and deletions, and a knock-in (KI) that harbored the precise substitution with the single-stranded oligodeoxynucleotide (ssODN). In PGN2, two pups out of five were genome-edited and harbored MU in the gene. We were able to obtain a single MU mouse from the KJR strain and a single KI mouse from the MSM strain, even though only one pup was obtained in each strain. Overall, around 59% of the total pups had genome-edited allele(s), with the ratio of KI to MU being 13:23 (Table 3).

### Application of i-GONAD with reduced number of transfer pulses

In the application of *i*-GONAD method with TP:6 condition, we were able to generate genome-edited mice in five wild strains, but the efficiencies of obtaining pups were extremely low in seven of the wild strains, including PGN2, NJL, HMI, SWN, KJR, MSM, and CHD. Particularly, no pup was obtained in four of the wild strains: CHD, HMI, NJL and SWN. To improve the pregnancy rate, we reduced the number of the transfer pulses from 6 to 3, in order to reduce the damage to the fertilized eggs. We applied the modified electroporation with TP:3 to the 7 wild strains that showed low pregnancy rates (< 20%), upon application of the *i*-GONAD with TP:6 method. Using the modified method, we were able to obtain newborn pups from six wild strains, out of the seven, with improved pregnancy rates in these strains (Fig. 3A and Table 4). In particular, the pregnancy rate of the MSM strain was higher by approximately sevenfold than that obtained using the *i*-GONAD with TP:6 method. As shown in Table 4, the *i*-GONAD with TP:3 method enabled to increase the pregnancy rates to more than 20% in six strains, although the HMI strain still exhibited a zero pregnancy rate. The efficiency of genome editing in the pups obtained using the *i*-GONAD with TP:3 method was lower (approximately 49% in total) as compared to that obtained using the *i*-GONAD with TP:6 method (approximately 62% in total). Genome sequences were analyzed for genomic mutations in each pup (Supplementary materials S2). The ratio of KI to MU was equal (11:11), which was higher than that obtained using the *i*-GONAD with TP:6 method. In the NJL strain, four pups were obtained only from one female, but none of the pups had genome-edited genes. Only in the HMI strain, we were unable to obtain any pups in either experiment of *i*-GONAD with different number of transfer pulses.

**Table 4:** Efficiencies of genome editing upon application of *i*-GONAD with 3 transfer pulses (TP:3).

Origin	Strain	Number of females	Pregnancy	Number of pups	Pups/female	MU	KI	Genome-editing efficiency
Laboratory	B6	1	1	3	3	na	na	na
Wild	PGN2	22	5 (22.7%)	12	0.55	3	1	4/11 (36.4%)
	BFM/2	N.A	N.A	N.A	N.A	N.A	N.A	N.A
	NJL	11	1 (9.1%)	4	0.36	0	0	0/4 (0.0%)
	BLG2	N.A	N.A	N.A	N.A	N.A	N.A	N.A
	CHD	11	3 (27.3%)	3	0.27	1	0	1/3 (33.3%)
	SWN	10	2 (20.0%)	5	0.50	1	2	3/5 (60.0%)
	KJR	10	3 (30.0%)	7	0.70	3	1	4/7 (57.1%)
	MSM	12	6 (50.0%)	16	1.33	3	7	10/15 (66.7%)
	HMI	11	0 (0.0%)	0	0.00	N.A	N.A	0/0 (0.0%)

*N.A*. not analyzed.

## Discussion

In this study, we reported that application of CRISPR/Cas9-genome editing using *i*-GONAD method is successful in most of the wild strains of Mishima battery, that is, seven out of the nine wild strains. Although the initial attempt of generating genome-edited mice using *i*-GONAD with 6 transfer pulses was efficient only in two wild strains, the efficiencies of generating genome-edited mice were very low in the other seven strains. However, the efficiencies were much improved upon application of electroporation with 3 transfer pulses, in five out of seven wild strains, which had exhibited low efficiencies in the 6 transfer pulses method. The results indicated that fertilized eggs from many wild strains, PGN2, NJL, CHD, SWN, KJR, MSM, and HMI were more sensitive to electroporation stimuli than those from the BFM/2 and BLG2 strains, as well as, the laboratory strain B6. Highly improved efficiencies of obtaining pups from each female (pups/female) upon applying *i*-GONAD with reduced number of electroporation stimuli in PGN2, NJL, CHD, SWN, KJR, and MSM suggested that there is a possibility to improve *i*-GONAD for wild strains, by modifying the electroporation condition to a milder one. Because no genome-edited mouse was obtained in the HMI and NJL strains in our study, reducing the number of the transfer pulses from three to lower numbers or reducing the voltage of the poring pulse from 40 to 30 V or even lower might be worth considering in the future studies attempting to carry out genome editing in these strains^36^. Among the nine wild strains, two strains, BFM/2 and BLG2, were efficiently genetically edited even upon use of 6 pulses, and their offspring were obtained with high efficiency using IVF. From these results, it should be emphasized that these two wild strains are suitable for gene modification using both *i*-GONAD and microinjection methods, at a level similar to that seen in B6.

We have also found that reducing the number of pulses resulted in reduced efficiencies of genome editing: 62% using 6 transfer pulses against 49% using 3 transfer pulses. The trade-off between the number of pulses and efficiency of genome editing is important to determine the condition of electroporation for *i*-GONAD. However, because genome editing efficiencies are relatively high upon using CRISPR/Cas9, *i*-GONAD could serve to be advantageous even if it leads to lower genome editing efficiencies because of application of lower number of pulses or lower voltage of poring pulses.

Genome editing using CRISPR/Cas9 is a highly efficient method to generate genetically manipulated mice^37–40^. Owing to the ease of preparation of gRNA and ssODN, this method allows to arrange for genome editing in a relatively short period of time, that is a total time of one week from the design of oligonucleotides to manipulation of the fertilized eggs. Even with the short period for the preparation, in vitro manipulation of fertilized eggs and microinjection into pronuclei require considerable experience and a proficient technique for handling of the eggs. In contrast, *i*-GONAD does not require ex vivo manipulation and microinjection, but only requires the technique of surgery to expose the oviduct out of the dorsal skin and apply electroporation on it, followed by suture surgery. Because of the low needs of this simple genome editing technique, it can be introduced widely in many laboratories.

Wild strains or wild mice exhibit behavioral differences, as compared to laboratory strains. Wild strains have attracted the attention of many researchers, due to their high level of inter-strain genetic variation, in addition to the phenotype maintained in them from wild mice. However, since the application of genetic engineering technology is not easy in these strains, congenic strains have been established to study the role of genes in the genetic background of wild mice^41^. In a study, a mouse with the transient receptor potential cation channel subfamily C member 2 gene, *TrpC2* (expressed in the vomeronasal organ), knocked out under the genetic background of C57BL/6J x 129S1/Sv, was backcrossed onto a wild mouse outbred stock, following which its behavioral phenotype was analyzed. The results showed that there was repressed aggressive behavior of the wild-backcrossed *TrpC2*^-/-^ mutant females toward another female, as compared to that seen in the *TrpC2*^+/+^ mice; however, differences between the genotypes were not observed at the laboratory genetic background. Similarly, aggressive behavior of females toward pups was intense and observed in 60% of females tested to be wild-backcrossed *TrpC2*^+/+^ congenic mice, while the aggression decreased in the wild-backcrossed *TrpC2*^-/-^ mutant females, despite no difference observed in their laboratory genetic background. These results suggested that studying the roles of genes under the genetic background of wild mice is a useful way to investigate gene functions from different aspects. However, making congenic mice by backcrossing with wild mice is a lengthy and laborious process that is not feasible for large-scale studies. Therefore, the generation of genome-edited mice using CRISPR/Cas9 technology with the *i*-GONAD method is highly effective for the study the roles of genes under the wild mouse genetic background.

Most of the wild strains still exhibit high level of wildness and low level of tameness to humans. A successful attempt has been made to generate mice stock that exhibit higher tameness, by means of selective breeding for tameness to humans^27^. In the experiment, a heterogeneous stock was first generated by crossing eight wild strains, PGN2, BFM/2, NJL, BLG2, CHD, KLR, MSM, and HMI, to expand the heterogeneity in the population with a huge number of combinations of different alleles. Applying selection on behavior for motivation to approach humans, active tameness, for seven to nine generations, resulted in a difference in the tameness scores of the selected groups, as compared to those of the non-selected control groups. Further genetic analysis and transcriptome analyses found several candidate genes for tameness, such as *Slc6a4*, *Oxtr*, etc*.*^27,42^. The limitation with using heterogeneous stock was a lack of a method for genetic engineering in the genetically heterogeneous population, because the mice group is an outbred stock and is derived from eight wild strains. High efficiencies for genome editing using the *i*-GONAD methodology described in this paper will be also useful to address the gene associated with tameness in the heterogeneous stock. Applying *i*-GONAD for multiple females will be able to generate a genome-edited outbred stock with genetic heterogeneity, which is similar to the wild mouse population.

In summary, in the present study, we have shown that the *i*-GONAD method is highly useful for genome editing in a series of wild strains. Although genome-edited mice were not obtained in two strains out of the nine wild strains, there is still a possibility to improve the rate of pup-birth by applying milder electroporation conditions. These results will open avenues to study genetic basis of many phenotypes that are found in wild strains. In addition, the method could serve to be useful for other mouse resources, in which genetic manipulation is difficult to achieve using the current microinjection method.

## Methods

### Mice

A laboratory strain B6 was purchased from CLEA Japan Inc. (Tokyo, Japan), while nine wild strains (BFM/2, BLG2, CHD, HMI, KJR, MSM, NJL, PGN2, and SWN) were bred at NIG (Shizuoka, Japan) (Table 1). All mice were maintained at the animal facility in NIG, under specific-pathogen-free conditions and controlled lighting conditions (daily light period: 06:00 to 18:00). The mice were kept in a temperature-controlled room (23 °C ± 2 °C) and food and water were provided ad libitum.

In the egg transfer experiments, female B6C3F1 were obtained by means of crosses between female B6 mice and male C3H/HeNJcl mice (CLEA Japan Inc.), and then used to serve as psuedopregnant recipients that were generated following mating with vasectomized male ICR mice (CLEA Japan Inc.).

### IVF experiments

Unfertilized eggs for the IVF experiments were collected from female mice (over 8 weeks of age) of one laboratory strain (B6) and nine wild strains. In the IVF experiments for each strain, 10 females were used in total and IVF operations were performed twice, with five females each. Female mice were superovulated by means of intraperitoneal injection of 5 IU pregnant mare serum gonadotropin (Serotropin, ASKA Animal Health, Tokyo, Japan), followed 48 h later by injection of 5 IU human chorionic gonadotropin (Gonatropin, ASKA Animal Health). About 17 h after the gonatropin injection, eggs were collected from the oviduct ampulla and incubated in human tubal fluid (HTF, ARK Resource, Kumamoto, Japan) media, at 37 °C, in an atmosphere with 5% CO_2_. In each experiment, the oocytes were collected from five superovulated females in each strain and all oocytes were incubated in one media, followed by counting of the number of oocytes for five females each time. Sperm were obtained from male mice (over 9 weeks of age) of the same strain as that from which eggs were collected and incubated in HTF media, at 37 °C for 1 h. Immediately after obtaining the eggs, the sperm were introduced into a drop of HTF media containing eggs, and the drop of HTF media was incubated at 37 °C, in an atmosphere with 5% CO_2_. Four to five hours after insemination, the fertilized eggs were moved to a fresh drop of HTF media and washed to remove cumulus cells and sperm by means of pipetting. After washing, the fertilized eggs were moved to drops of potassium-supplemented simplex optimized medium (KSOM, ARK Resource) media and incubated at 37 °C in an atmosphere with 5% CO_2_, overnight. Normally developed 2-cell embryos, abnormal cells, and unfertilized eggs were observed and documented using the camera in an inverted microscope (IX71, Olympus, Tokyo, Japan), following which the 2-cell embryos were transferred into the oviducts of pseudopregnant B6C3F1 females.

### CRISPR reagents

The CRISPR gRNAs, which were used to perform genome editing using the CRISPR/Cas9 system on the *Adcyap1* gene that encodes the neuropeptide PACAP, were designed using CRISPOR^43^, followed by CRISPR-Cas9 guide RNA design checker (https://sg.idtdna.com/site/order/designtool/index/CRISPR_SEQUENCE) developed by Integrated DNA Technologies (Coralville, IA, USA). No polymorphism in the nine wild strains and B6 was found in the designed gRNA sequences by MoG+ , Mouse Genome Database with high added values (https://molossinus.brc.riken.jp/mogplus/#JF1). The gRNAs were generated as follows:

*STEP 1* The DNA fragment of Cas9 tracrRNA scaffold was PCR-amplified with a primer set (gRNA-F/T7-gRNA-PCR-R, Supplementary materials S1), using Tks Gflex DNA Polymerase (Takara Bio, Shiga, Japan) and DR274 plasmid (plasmid number 42250, Addgene, Watertown, MA, USA) linearized using *Dra*I (Nippon Gene, Tokyo, Japan) as the template.

*STEP 2* Two DNA fragments containing T7 promoter and gRNA scaffold, PACAP-ex3-52fw and PACAP-ex3-56fw, were synthesized using the purified-PCR product amplified in STEP 1 as the template and KOD-Plus-Neo (Toyobo, Osaka, Japan) as well as the primer sets, PACAP-ex3-52fw/T7-gRNA-PCR-R and PACAP-ex3-56fw/ T7-gRNA-PCR-R, respectively (Supplementary Materials S1). The two DNA fragments used for guide RNA synthesis were purified using FastGene® Gel/PCR Extraction Kit (Nippon Genetics, Tokyo, Japan).

*STEP 3* Two gRNAs were in vitro transcribed using two purified DNA fragments as templates, with the MEGAshortscript™ T7 Transcription Kit (Thermo Fisher Scientific, Waltham, MA, USA). The two gRNAs were purified using the MEGAclear™ Kit (Thermo Fisher Scientific).

All primers were commercially synthesized by Thermo Fisher Scientific. The ssODN, PACAP_Larm-3xSTOP-spA-Rarm, which has three stop codons in three coding frames followed by a poly-A signal was commercially synthesized by Eurofins Genomics (Tokyo, Japan) and used as a template for homology-directed repair, to generate mice carrying the edited gene for PACAP (Supplementary materials S3).

### Preparation of electroporation solution

The electroporation solution contained two in vitro synthesized gRNAs, a commercially purchased Alt-R S.p. Cas9 Nuclease V3 protein (Integrated DNA Technologies), and a ssODN. The final concentrations of the components were 1 µg/µl (Cas9 protein), 1 µg/µl (each gRNA), and 2 µg/µl (ssODN). The solution was diluted using Opti-MEM (Thermo Fisher Scientific) to up to 3 µl per one subject female mouse. These components were mixed on ice. The solution was heated at 37 °C for 10 min before use.

### i-GONAD procedure

Genome editing using the *i*-GONAD method was conducted based on previous reports^33,36^. Female mice over 8 weeks of age were co-housed with adult male mice of the same strain, at a time between 16:00 and 18:00, and kept until the next day. Copulation plugs were confirmed by means of visual inspection the next morning, between 9:00 and 10:00. Females that displayed a vaginal plug were used for electroporation experiments on the same day. Surgical procedures were performed between 15:00 and 18:00. The females that displayed a plug upon copulation were anesthetized using 2–4% isoflurane (Pfizer Japan, Tokyo, Japan) using an inhalation anesthesia system (Narcobit-E II, Natsume Seisakusho, Tokyo, Japan) and placed on a heating-plate at 37 °C. Their dorsal skin was severed using scissors, following which the ovary, oviduct, and part of the uterus were exposed to the outside of the dorsal skin. Approximately 1.0–1.5 µl of electroporation solution was injected into the oviduct lumen upstream of the oviduct ampulla, using a micropipette, under observation in a stereo microscope (SZX7, Olympus). After injection, the oviduct and its ampulla were covered with a piece of wet paper (Kimwipe, Nippon Paper Crecia, Tokyo, Japan) dipped in phosphate-buffered saline (PBS) and grasped with tweezer-type electrodes (CUY652P 2.5 × 4, Nepa Gene, Chiba, Japan) dipped in PBS. Electroporation was performed using the electroporator NEPA21 (Nepa Gene). The parameters for electroporation were as follows: poring pulse: 40 V, 5-ms pulse, 50-ms pulse interval, 3 pulses, 10% decay, ± polarity, and transfer pulse: 10 V, 50-ms pulse, 50-ms pulse interval, 6 pulses, 40% decay, ± polarity. Wild strains that displayed a low rate of pregnancy (< 20%) in the first series of electroporation experiments were used for the next series of *i*-GONAD experiments with modified electroporation pulses, from 6 to 3, in terms of transfer pulses. No other parameter was changed in this experiment. After electroporation, the ovary, oviduct, and part of the uterus were returned to under the skin, and the severed site was sutured using Wound Clip (Becton Dickinson, Franklin Lakes, NJ, USA). After 20 d, the subject mice were checked for presence of fetuses.

### Analysis of i-GONAD efficiency

Genomic DNA was extracted from a piece of ear sample in each progeny. Each ear piece was put into a 1.5 ml tube with 100 µl of TE buffer (1 M Tris–Hcl, 0.5 M ethylenediaminetetraacetic acid (EDTA), pH 8.0), and the sample tube was heated at 95 °C for 5 min. After the sample returned to room temperature, 2 µl of 10 mg/ml Proteinase K (Roche, Basel, Switzerland) was added to each tube and incubated overnight at 55 °C. After the incubation, the samples were heated at 95 °C for 5 min and the solutions were directly used for PCR amplification, as template DNA. PCR amplification for target loci was performed in a total solution of 10 µl, containing 5 µl of 2 × Buffer for KOD FX Neo (Toyobo), 2 µl of 2 mM deoxyribonucleoside-triphosphates (dNTPs), 0.3 µl of each primer (PACAP-ex3-left F1 and PACAP-ex3-right R1) (Supplementary materials S1), 0.05 µl of KOD FX Neo, 1.35 µl of H_2_O, and 1 µl of template DNA, using the following conditions: denaturation at 94 °C for 2 min, 35 cycles of 98 °C for 10 s, 60 °C for 30 s, 68 °C for 30 s, and an extension at 68 °C for 1 min. DNA products were purified using the FastGene® Gel/PCR Extraction Kit (Nippon Genetics). Purified DNA products were examined using 3% agarose gel electrophoresis, following which the amplicons with mutant alleles were sequenced. The sequence analyses helped clarify whether the genome editing sites were knocked in precisely or mutated with unexpected sequence changes. Sequences of the PCR products were generated by Eurofins sequencing service.

### Graphs and figures

All graphs and illustrations were generated using Prism 9 (GraphPad Software Inc., San Diego, CA, USA) and BioRender (https://biorender.com), respectively.

### Institutional review board statement

The study was conducted according to the guidelines of the ARRIVE and the institutional guideline, and were approved by the Institutional Animal Care and Use Committee at NIG (permit numbers 31-7, 31-8, R2-13, and R2-15).

## Supplementary Information

Supplementary Information.

## Data Availability

The datasets generated and/or analysed during the current study are available in DDBJ, the DNA Data Bank of Japan (http://getentry.ddbj.nig.ac.jp/top-e.html), Accession numbers, LC708315–LC708363 (Supplementary materials S1 and S2).
