# Author Correction: Efficient genome editing in wild strains of mice using the *i*-GONAD method

**DOI:** 10.1038/s41598-023-30538-7

**Published:** 2023-02-28

**Authors:** Yuji Imai, Akira Tanave, Makoto Matsuyama, Tsuyoshi Koide

**Affiliations:** 1 Mouse Genomics Resource Laboratory, National Institute of Genetics, Mishima, 411-8540 Japan; 2 Laboratory for Mouse Genetic Engineering, RIKEN Center for Biosystems Dynamics Research, Osaka, 565-0871 Japan; 3 Division of Molecular Genetics, Shigei Medical Research Institute, Okayama, 701-0202 Japan; 4 Department of Genetics, SOKENDAI (The Graduate University for Advanced Studies), Mishima, 411-8540 Japan

Correction to: *Scientific Reports* https://doi.org/10.1038/s41598-022-17776-x, published online 15 August 2022.

The original version of this Article contained an error in Figure 3, panel A, where the number of B6 mice “(6, 1)” was incorrectly given as “(6, 22)”. The original Figure 3 and accompanying legend appear below.

**Figure 3:**
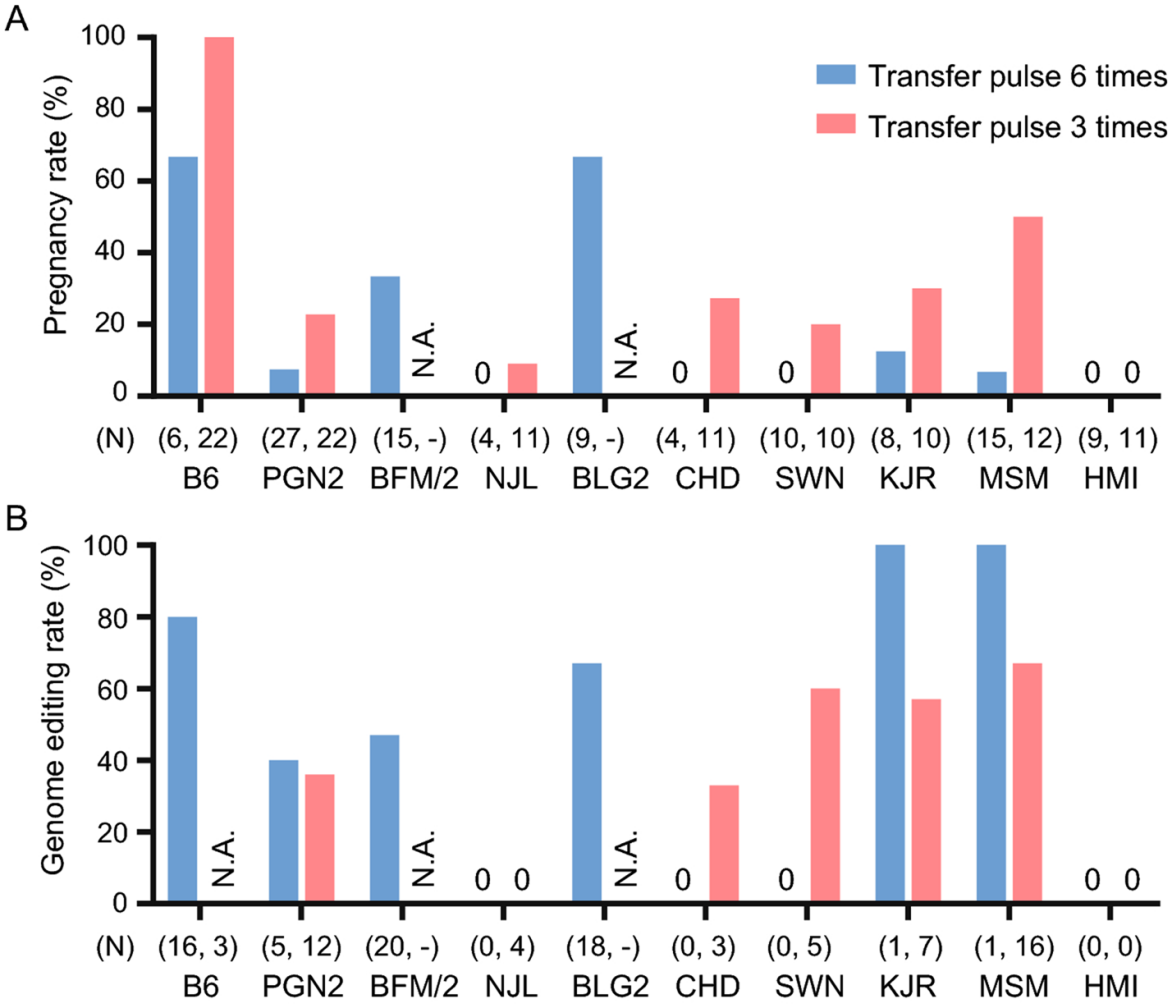
Comparison of efficiencies between the methodologies of 6 times transfer pulse (TP:6) and 3 times transfer pulse (TP:3) in the *i*-GONAD experiments. N; number of pups obtained in the *i*-GONAD experiments in each strain. (**A**) Overview of the pregnancy rates between TP:6 and TP:3. N.A., not analyzed. (**B**) Efficiencies of genome editing upon application of TP:6 and TP:3. The efficiencies were calculated by dividing the number of genome-edited pups by the number of all the sampled pups in each strain. The B6 strain and seven wild strains that displayed low pregnancy rates (< 20%) were further examined with the TP:3 condition.

The original Article has been corrected.

