# Supplementary material for: Efficient genome editing in wild strains of mice using the i-GONAD method
Source: Sci Rep. 2022 Aug 15;12:13821. doi: 10.1038/s41598-022-17776-x (PMC9378668; doi:10.1038/s41598-022-17776-x)
Supplement: Supplementary file 1 — Supplementary Information. [file 41598_2022_17776_MOESM1_ESM.pdf]

# Efficient genome editing in wild strains of mice using the *i*-GONAD method

**Yuji Imai, Akira Tanave, Makoto Matsuyama, and Tsuyoshi Koide**

## Supplementary materials

### S1. Sequences of genome editing sites for pups born with transfer pulse 6 times (TP:6)

The sequences highlighted with yellow indicate exon sequence, the sequences highlighted with red indicate stop codons, and blue indicate polyA-signal sequence. Texts in red indicate unexpected insertion and hyphens indicate unexpected deletion.

| strain | No. | genotype | sequence | Accession No. |
|---|---|---|---|---|
| B6 | 946-1 | mosaic | | |
| | 946-2 | w | GACGAGGCTTACGACCAGGACGGAAACCCG | LC708315 |
| | | -4 | GACGAGGC----GACCAGGACGGAAACCCG | LC708316 |
| | 946-3 | w | GACGAGGCTTACGACCAGGACGGAAACCC | |
| | | +1 | GACGAGGCTTACGCACCAGGACGGAAACCC | LC708317 |
| | 947-1 | mosaic | | |
| | 947-2 | w | CTTACGACCAGGACGGAAACCCG | |
| | | KI | CTTACTGACTAGCTAATAAAAGATCTTTATTTTCATTAGATCTGTGTGTTGGTTTTTTGTGTGGACC | LC708318 |
| | 947-3 | mosaic | | |
| | 947-4 | KI | GGCTTACTGACTAGCTAATAAAAGATCTTTATTTTCATTAGATCTGTGTGTTGGTTTTTTGTGTGGACCAGGACG | LC708319 |
| | | KI | GGCTTACTGACTAGCTAATAAAAGATCTTTATTTTCATTAGATCTGTGTGTTGGTTTTTTGTGTGGACCAGGACG | LC708319 |
| | 1130-1 | -2 | TTAC--CCAGGACGGAAACCC | LC708320 |
| | | undecidable | TTACGACCAGCGGCTTTCCGTCCTGGTC | |
| | 1130-2 | KI, mosaic | | |
| | 1130-3 | mosaic | | |
| | 1142-1 | w | | |
| | 1142-2 | w | | |
| | 1142-3 | mosaic | | |
| | 1142-4 | KI | GGCTTACTGACTAGCTAATAAAAGATCTTTATTTTCATTAGATCTGTGTGTTGGTTTTTTGTGTGGACCAGGACG | LC708321 |

| | | | | |
|---|---|---|---|---|
| | | KI | GGCTTACTGACTAGCTAATAAAAGATCTTTATTTTCATTAGATCTGTGTGTTGGTTTTT TGTGTGGACCAGGACG | LC708321 |
| | 1142-5 | w | | |
| BLG2 | 948-1 | KI, | TTACTGACTAGCTAATAAAAGAT | LC708322 |
| | | -95 | TT-(-93)-TACTATCCAGCGGACAGGA | LC708323 |
| | 948-2 | KI | TTACTGACTAGCTAATAAAAGA | LC708324 |
| | | -3 | TTAC---CAGGACGGAAACCCGCTG | LC708325 |
| | 948-3 | Patial KI | GAGGCTTTGTTGGTTTTTTGTGTGGACCAGGAC | LC708326 |
| | | -3 | GAGGC---CGACCAGGACGGAAACCCGCTGCAAGAC | LC708327 |
| | 949-1 | mosaic | | |
| | 949-2 | mosaic | | |
| | 949-3 | -3 | TTAC---CAGGACGGAAACCCGCTGCAAGACT | LC708328 |
| | | -3 | TTAC---CAGGACGGAAACCCGCTGCAAGACT | LC708328 |
| | 991-1 | KI | TTACTGACTAGCTAATAAAAGATCT | LC708329 |
| | | -4 (-5, +1) | TTA----AAGGACGGAAACCC | LC708330 |
| | 991-2 | w | | |
| | 991-3 | w | | |
| | 991-4 | w | | |
| | 991-5 | w ,mosaic | | |
| | 991-6 | w, KI, mosaic | | |
| | 991-7 | w | GACGAGGCTTACGACCAGGACGGAAACCCG | |
| | | -3 | GACGAGGCTTAC---CAGGACGGAAACCCGCTGCAA | LC708331 |
| | 991-8 | w | | |
| | 991-9 | w, mosaic | | |
| | 991-10 | KI | GGCTTACTGACTAGCTAATAAAAGATCTTTATTTTCATTAGATCTGTGTGTTGGTTTTT TGTGTGGACCAGGACG | LC708332 |
| | | Patial KI | GGCTTCTGTGTGTTGGTTTTTTGTGTGGACCA | LC708333 |
| | 991-11 | w | | |
| | 991-12 | w | | |
| KJR | 950-1 | w, mosaic | | |
| MSM | 950-2 | KI | GGCTTACTGACTAGCTAATAAAAGATCTTTATTTTCATTAGATCTGTGTGTTGGTTTTT TGTGTGGACCAGGACG | LC708334 |
| | | mosaic | | |
| PGN2 | 975-1 | mosaic | | |

| | | | | |
|---|---|---|---|---|
| | 975-2 | w | | |
| | 975-3 | mosaic | | |
| | 975-4 | w | | |
| | 1040-1 | w | | |
| BFM/2 | 981-1 | w | ttttaatgaccctccttttggctgtcccgcagACCAGAAGACGAGGCTTACGACCAGGACGGAAACCCG | |
| | | -55(-56, +1) | ttttaa-------------------------------------------------------AAAACCCG | LC708335 |
| | 981-2 | w | | |
| | 981-3 | w | | |
| | 981-4 | Patial KI | GGCTTCTGTGTGTTGGTTTTTTGTGTGG | LC708336 |
| | | -3 | GAGGCTT---ACCAGGACGGAAACCCGCTGCAA | LC708337 |
| | 981-5 | w | TTACGACCAGGACGGAAACCCG | |
| | | -6 | TTACGAC------GGAAACCCG | LC708338 |
| | 982-1 | w | TTACGACCAGGACGGAAACCCGCTGC | |
| | | -15 | TTAC---------------CCGCTGC | LC708339 |
| | 982-2 | w | | |
| | 982-3 | w | | |
| | 982-4 | w | | |
| | 982-5 | w | | |
| | 982-6 | w, mosaic | | |
| | 1033-1 | w | | |
| | 1033-2 | w | GACGAGGCTTACGACCAGGACGGAAACCCGCTGC | |
| | | -18 | GACG------------------GAAACCCGCTGC | LC708340 |
| | 1033-3 | w | | |
| | 1103-1 | w | | |
| | 1103-2 | -20 | gACCAG--------------------GACGGAAACCCGCTGC | LC708341 |
| | | mosaic | | |
| | 1103-3 | KI (-8del) | ttggc--------agACCAGAAGACGAGGCTTACTGACTAGCTAATAAAA | LC708342 |
| | | +1(+2, -1) | ttggctgtcccgcagACCAGAAGACGAGGCTTACGACCAACGAC | LC708343 |

**S2. Sequences of genome editing sites for pups born with transfer pulse 3 times (TP:3)**

The sequences highlighted with yellow indicate exon sequence, the sequences highlighted with red indicate stop codons, and blue indicate polyA-signal sequence. Texts in red indicate unexpected insertion and hyphens indicate unexpected deletion.

| strain | No. | genotype | sequence | Accession No. |
|---|---|---|---|---|
| MSM | 1104-1 | w | | |
| | 1104-2 | -2 | GGCTTA--ACCAGGACGGAAACCCGCTGCAAG | LC708344 |
| | | -2 | GGCTTA--ACCAGGACGGAAACCCGCTGCAAG | LC708344 |
| | 1104-3 | KI | GACGAGGCTTACTGACTAGCTAATAAAAGAT | LC708345 |
| | | -1 | GACGAGGCTTAC-ACCAGGACGGAAACCCGCT | LC708346 |
| | 1104-4 | KI | GAGGCTTACTGACTAGCTAATAAAAGATCTTT | LC708347 |
| | | KI | GAGGCTTACTGACTAGCTAATAAAAGATCTTT | LC708347 |
| | 1105-1 | KI | GAGGCTTACTGACTAGCTAATAAAAGATCTTT | |
| | | -25 | gcag-------------------------GACGGAAACCCGCTGCAAGAC | LC708348 |
| | | -3 | GAGGCTTAC---CAGGACGGAAACCCGCTGC | LC708349 |
| | 1105-2 | w | | |
| | 1116-1 | w | | |
| | 1125-1 | KI, mosaic | | |
| | 1125-2 | w | | |
| | 1126-1 | KI | GAGGCTTACTGACTAGCTAATAAAAGATCTTT | LC708350 |
| | | w | GACGAGGCTTACGACCAGGACGGAAACCCGCTGC | |
| | | -18 | GACG------------------GAAACCCGGCTGC | LC708351 |
| | 1126-2 | KI | GAGGCTTACTGACTAGCTAATAAAAGATCTTT | LC708352 |
| | | w | GACGAGGCTTACGACCAGGACGGAAACCCGCTGC | |
| | | -1 | TTAC-ACCAGGACGGAAACCC | LC708353 |
| | 1126-3 | KI | TTACTGACTAGCTAATAAAAGATCTTT | LC708354 |
| | | -25 (-26, +1) | TTACGACCT--------------------------TATGACTGGGACCC | LC708355 |
| | 1127-1 | w | GAGGCTTACGACCAGGACGGAAACCCGCTGCAAG | |
| | | (-11, 1 substitution) | GAGG-----------GACGGATACCCGCTGCAAG | LC708356 |
| | 1127-2 | w, mosaic | | |
| | 1127-3 | w | | |
| PGN2 | 1149-1 | w | | |
| | 1149-2 | w | | |

| | | | | |
|---|---|---|---|---|
| | | +2 | TTACGGGACCAGCACGGAAACCCG | LC708357 |
| | 1149-3 | -3 | TTAC- - -CAGGACGGAAACCCG | LC708358 |
| | 1167-1 | w | | |
| | 1168-1 | w | | |
| | 1168-2 | PolyA KI (without stop codons) | GGCTAATAAAAGATCTTTATTTCATTAGA | LC708359 |
| | | mosaic | | |
| | 1168-3 | w | | |
| | 1172-1 | w, mosaic | | |
| | 1262-1 | w, -12, mosaic | | |
| | 1273-1 | w | | |
| | 1273-2 | w | | |
| KJR | 1169-1 | w, KI, mosaic | | |
| | 1169-2 | w | | |
| | 1190-1 | w | | |
| | 1190-2 | w, mosaic | | |
| | 1207-1 | w, mosaic | | |
| | 1208-1 | w | | |
| | 1208-2 | w, mosaic | | |
| NJL | 1162-1 | w | | |
| | 1162-2 | w | | |
| | 1162-3 | w | | |
| | 1162-4 | w | | |
| CHD | 1209-1 | w, mosaic | | |
| | 1234-1 | w | | |
| | 1234-2 | w | | |
| SWN | 1337-1 | w | | |
| | 1337-2 | w | | |
| | 1352-1 | -3 | GAGGCTTAC- - -CAGGACGGAAACCCGC | LC708360 |
| | | -13 | GAGGC- - - - - - - - - - - - -GGAAACCCGC | LC708361 |
| | 1352-2 | Partial PolyA (+3) | TTACGACCATTAGATCTGTGTGTTGGTTTTTTGTGTGGACCA | LC708362 |
| | | Partial PolyA (+3) | TTACGACCATTAGATCTGTGTGTTGGTTTTTTGTGTGGACCA | LC708362 |
| | 1352-3 | Partial PolyA (+1) | TTACGACGTCATTAGATCTGTGTGTTGGTTTTTTGTGTG | LC708363 |
| | | w | TTACGACCAGGACGGAAACCCGCTGCAA | |

**Supplementary materials S3. Oligonucleotides used in this study**

**gRNA-F** (GTTTTAGAGCTAGAAATAGC)

**T7-gRNA-PCR-R** (AAAAAAGCACCGACTCGGTGCC)

**PACAP-ex3-52fw**
(GGATCCTAATACGACTCACTATAGGGAGAAGACGAGGCTTACGACCGTTTTAGAGCTAGAAATAGC)

**PACAP-ex3-56fw**
(GGATCCTAATACGACTCACTATAGGGGACGAGGCTTACGACCAGGAGTTTTAGAGCTAGAAATAGC)

**PACAP_Larm-3xSTOP-spA-Rarm**
(ggctgtcccgcagACCAGAAGACGAGGCTTACTGACTAGCTAATAAAAGATCTTTATTTTCATTAGATCTGTGT GTTGGTTTTTTGTGTGGACCAGGACGGAAACCCGCTGCAAGACTTCTA)

**PACAP-ex3-left F1** (GACCATTCTGGAAGTAAGGGCAAT)

**PACAP-ex3-right R1** (GTTGTACCTCCTGTCCGCTGGAT)
